# Clinical utility of optical scatter-derived red blood cell parameters for the rapid identification and correction of lipemic interference

**DOI:** 10.3389/fmed.2026.1835266

**Published:** 2026-06-25

**Authors:** Xinjuan Zhang, Jiling Zhang, Yandan Wu

**Affiliations:** Beijing Shunyi District Hospital, Shunyi Teaching Hospital of Capital Medical University, Beijing, China

**Keywords:** Delta-HGB, hemoglobin, lipemia, optical scatter method, plasma replacement

## Abstract

**Background:**

Lipemic interference is a common cause of spuriously increased hemoglobin (HGB) and red blood cell indices in routine complete blood count testing. This study evaluated whether Delta-HGB, an optical scatter-derived parameter generated in the reticulocyte (RET) channel of an automated hematology analyzer, can identify lipemic interference and whether optical scatter-derived HGB-O and MCHC-O can rapidly correct lipemia-related bias.

**Methods:**

A total of 471 complete blood count specimens collected between April 2025 and February 2026 were included, comprising 158 non-lipemic and 313 lipemic samples. The precision and linearity of optical scatter-based HGB measurement were first assessed. HGB-I obtained by the conventional colorimetric method was then compared with HGB-O obtained by the optical scatter method in non-lipemic samples. Lipemic specimens were analyzed using the colorimetric method, optical scatter method, plasma replacement, and formula-based correction. Receiver operating characteristic (ROC) analysis was used to evaluate the performance of Delta-HGB and MCHC-I, and agreement among correction methods was assessed.

**Results:**

The optical scatter method showed acceptable within-run precision, with coefficients of variation of 1.0–1.3% in non-lipemic samples and 0.9–1.4% in lipemic samples, all meeting the WS/T 406–2012 criterion. Linearity was excellent. In non-lipemic samples, HGB results measured by the colorimetric and optical scatter methods did not differ significantly. For overall lipemic interference, the AUC was 0.703 (95% CI: 0.658–0.749) for Delta-HGB and 0.668 (95% CI: 0.620–0.716) for MCHC-I. After stratification by lipemic index, discrimination of severe lipemic interference improved markedly, with AUCs of 0.988 (95% CI: 0.970–1.000) and 0.979 (95% CI: 0.950–1.000), respectively. The optimal cutoffs were 7 g/L for Delta-HGB and 359.5 g/L for MCHC-I, both yielding sensitivity and specificity above 95%. Optical scatter-derived HGB, MCHC, and MCH showed good agreement with plasma replacement and formula-based correction.

**Conclusion:**

Delta-HGB combined with MCHC-I is useful for identifying severe lipemic interference. Optical scatter-derived parameters can correct lipemia-induced bias without specimen pretreatment, providing a rapid and practical strategy for routine laboratory use.

## Introduction

Colorimetric measurement is widely used in clinical laboratories for hemoglobin (HGB) determination. However, turbidity caused by lipid particles in lipemic specimens is a major source of falsely elevated HGB results and may also spuriously increase mean corpuscular hemoglobin (MCH) and mean corpuscular hemoglobin concentration (MCHC), with MCHC usually showing the most pronounced change (1–5). Accordingly, an abnormally increased MCHC is commonly regarded as an initial clue to lipemic interference (3–8). Nevertheless, elevated MCHC is not specific to lipemia, because cold agglutination and certain erythrocyte disorders can produce similar changes (3–5, 8–10). Reliance on MCHC alone may therefore lead to missed cases or misclassification.

Plasma replacement is generally considered a reference approach for correcting lipemia-induced HGB interference, but it requires centrifugation and replacement of the plasma fraction. In severely lipemic samples, repeated manipulation may be required, making the procedure cumbersome and time-consuming (11, 12). Formula-based correction has also been proposed and has shown good agreement with plasma replacement (7, 12, 13), but it still requires plasma separation and measurement of plasma HGB. Because complete blood count testing is usually performed directly on whole blood, lipemia is often difficult to recognize visually before centrifugation. A rapid method that can both identify lipemic interference and directly provide corrected results would therefore be of substantial practical value.

In the RET channel, the optical scatter method derives HGB-O using the following principle: CELLPACK DFL reagent creates pores in the erythrocyte membrane and Fluorocell RET stains the intracellular contents. The most frequent value of forward scatter from the mature red blood cell population (RBC-Y) is then converted into the red blood cell hemoglobin equivalent (RBC-He). Because RBC-He is highly correlated with MCH, it can substitute for MCH in the calculation of HGB-O, while simultaneously generating the derived parameter Delta-HGB (4, 12).

The present study aimed to evaluate the performance of Delta-HGB for identifying lipemic interference and to investigate the utility of HGB-O and related red blood cell parameters for correcting lipemia-induced bias, thereby providing a practical laboratory strategy for the rapid recognition and accurate reporting of lipemic samples.

## Materials and methods

### Study population

This retrospective study included 471 complete blood count specimens collected from outpatients, individuals undergoing physical examination, and inpatients at Shunyi Teaching Hospital of Capital Medical University between April 2025 and February 2026. Patients were 20–77 years old, including 284 men and 187 women. All specimens were analyzed within 4 h after collection. According to specimen lipemia status, samples were classified as non-lipemic or lipemic. The non-lipemic group comprised 158 specimens with triglyceride (TG) and total cholesterol concentrations within the reference intervals, whereas the lipemic group comprised 313 specimens with TG of 13.0 ± 10.4 mmol/L and total cholesterol of 6.6 ± 2.6 mmol/L. Samples were assigned to the non-lipemic group when triglyceride and total cholesterol concentrations were within the reference intervals, and to the lipemic group when visible or instrument-defined lipemia was present. Samples were excluded if they showed potential analytical or biological confounders that could affect hemoglobin measurement or erythrocyte indices, including hemolysis, icterus, cold agglutinins, hemoglobinopathies, marked red blood cell morphological abnormalities, or other red blood cell disorders. Samples with insufficient volume, clotting, delayed analysis beyond 4 h, or incomplete clinical laboratory data were also excluded.

The study used residual clinical samples and did not require additional blood collection. Ethical approval was granted by the Ethics Committee of Shunyi Teaching Hospital of Capital Medical University, and the requirement for informed consent was waived in accordance with relevant regulations for retrospective biomedical research using anonymized residual specimens.

### Instruments and reagents

Blood cell parameters were measured using the XN-9000 automated hematology analyzer (Sysmex, Kobe, Japan) with manufacturer-provided reagents and quality control materials. TG and total cholesterol were measured on the AU5800 chemistry analyzer (Beckman Coulter, Brea, CA, United States) using compatible GPO-PAP reagents (Beijing Zhongsheng Beikong Bio-Technology Co., Ltd., Beijing, China). Centrifugation was performed using a BY-600C tabletop centrifuge (Baiyang, Beijing, China). Plasma used for formula-based correction was collected in additive-free microcentrifuge tubes. All analyses were performed with the instruments under routine internal quality control.

### Study procedures

#### Precision and linearity of optical scatter-based HGB measurement

For within-run precision, three non-lipemic and three lipemic specimens were selected, with one low-, one medium-, and one high-HGB sample in each group. Each specimen was measured 11 consecutive times, and the final 10 results were used to calculate the coefficient of variation (CV).

For linearity assessment, one high-HGB sample (HGB = 210 g/L) was serially diluted with saline to 100, 80, 60, 40, 20, and 10% of the original concentration. Each level was measured in triplicate, and the mean value was used to evaluate the linear relationship between observed and expected values.

#### Comparison of optical scatter and colorimetric methods in non-lipemic samples

Under acceptable quality control conditions, 155 non-lipemic specimens were analyzed in both the routine complete blood count (CBC) mode and the reticulocyte (RET) channel. The conventional colorimetric HGB and associated erythrocyte parameters obtained in CBC mode were denoted HGB-I and MCHC-I, respectively, whereas RET channel measurements were denoted HGB-O and MCHC-O. Delta-HGB was calculated as HGB-I minus HGB-O. Differences, bias, and agreement between the two methods were evaluated.

#### Comparison of optical scatter, plasma replacement, and formula-based correction in lipemic samples

A total of 310 lipemic specimens were included in the method comparison analysis. Lipemic turbidity was graded using the semiquantitative HIL lipemic index obtained from biochemistry testing: lipemic index 1 was defined as 1+, indices 2–3 as 2+, and index > = 4 as 3+. On this basis, the lipemic samples were divided into a 1 + group (*n* = 116), a 2 + group (*n* = 112), and a 3 + group (*n* = 82).

For baseline and optical scatter measurements, each lipemic specimen was first analyzed in CBC mode to obtain the original values and then measured in the RET channel to obtain optical scatter-derived parameters, including Delta-HGB.

For plasma replacement, specimens were centrifuged at 1,500 x g for 10 min. The upper lipemic plasma layer was removed as completely as possible and replaced with an equal volume of the analyzer diluent. After thorough mixing, the sample was reanalyzed in CBC mode. To ensure procedural reliability, the bias in red blood cell count before and after plasma replacement was required to satisfy |RBC_replacement - RBC_original|/RBC_original x 100% < 3.0%. Plasma replacement as a commonly used practical reference approach for correcting lipemia-induced interference in complete blood count testing, rather than as a definitive gold standard.

For formula-based correction, the separated lipemic plasma obtained during plasma replacement was measured in CBC mode to determine plasma HGB. Corrected HGB was calculated as HGB_corrected = HGB_original - HGB_plasma x (1 - HCT_original). Corrected MCH and MCHC were subsequently derived from the corrected HGB values (Table 1).

**Table 1:** Three correction methods for HGB and related indices.

Parameter	Optical scatter method	Plasma replacement method	Formula-based correction method
HGB	HGB-O	HGB after replacement	HGB_original - HGB_plasma x (1 - HCT_original)
MCH	MCH-O (RBC-He)	HGB_after replacement/RBC_original	HGB_corrected/RBC_original
MCHC	MCHC-O	HGB_after replacement/HCT_original	HGB_corrected/HCT_original

### Outcome measures

The main study outcomes were as follows: (1) within-run precision and linearity of optical scatter-based HGB measurement; (2) differences and agreement between HGB-I and HGB-O as well as between MCHC-I and MCHC-O in non-lipemic samples; (3) the ability of Delta-HGB and MCHC-I to identify lipemic interference in lipemic samples; and (4) differences and agreement among the optical scatter method, plasma replacement, and formula-based correction for corrected HGB, MCHC, and MCH.

### Statistical analysis

Statistical analyses were performed using GraphPad Prism 8.0.2. Normally distributed continuous variables are presented as mean ± standard deviation and were compared using the paired t test. Non-normally distributed variables are expressed as median (P25, P75) and were compared using the Wilcoxon signed-rank test for two-group comparisons or the Kruskal-Wallis test for multiple-group comparisons, as appropriate. Agreement between methods was assessed using Bland–Altman analysis. Receiver operating characteristic (ROC) curves were used to evaluate the performance of Delta-HGB and MCHC-I for identifying lipemic interference, and the area under the curve (AUC) and optimal cutoffs were calculated. A two-sided *p* value < 0.05 was considered statistically significant.

## Results

### Precision and linearity of optical scatter-based HGB measurement

The optical scatter method demonstrated good within-run precision. In non-lipemic samples, the CVs for low-, medium-, and high-HGB specimens were 1.3, 1.0, and 1.0%, respectively. In lipemic samples, the corresponding CVs were 1.4, 0.9, and 1.0%. All values met the WS/T 406–2012 requirement for HGB precision (<=1.5%).

Linearity was excellent across the tested range, with a regression equation of Y = 1.0034X - 1.1274 and R^2 = 1.000, indicating satisfactory linear performance of the optical scatter method under the conditions of this study.

### Comparison of optical scatter and colorimetric methods in non-lipemic samples

**Figure 1:**
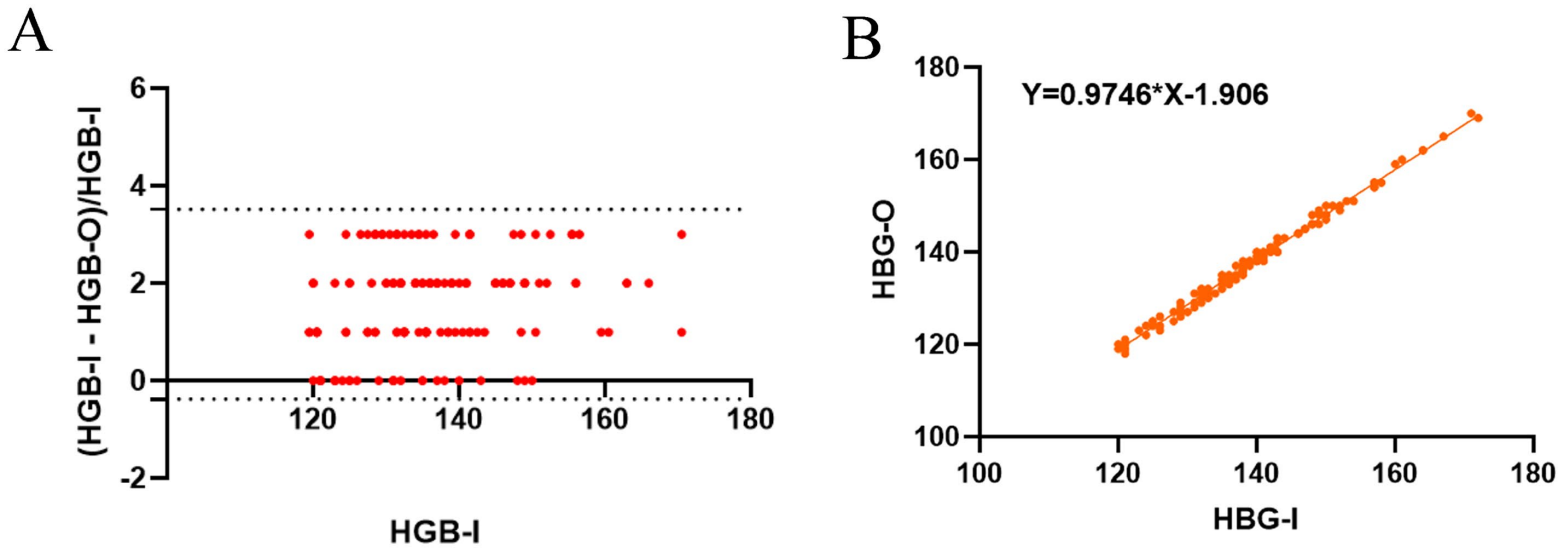
Correlation and agreement of hemoglobin measured by the colorimetric and optical scatter methods in non-lipemic samples. **(A)** Linear regression analysis between HGB-I and HGB-O. **(B)** Bland–Altman analysis of agreement between HGB-I and HGB-O.

Among 155 non-lipemic specimens, HGB measured by the optical scatter method did not differ significantly from that measured by the colorimetric method (*p* > 0.05). Bland–Altman analysis showed that the bias between the two methods was within an acceptable range, with 80% of biases below 3.5%. Linear regression analysis showed a strong correlation (*r* = 0.993), indicating good agreement between the two methods in non-lipemic samples (Figure 1).

### Comparison of Delta-HGB and MCHC-I between lipemic and non-lipemic groups and their ability to identify lipemic interference

**Figure 2:**
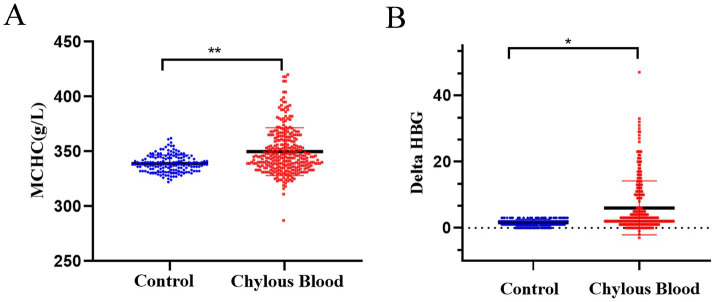
Comparison of Delta-HGB and MCHC-I between the non-lipemic and lipemic groups. **(A)** Comparison of MCHC-I between groups. **(B)** Comparison of Delta-HGB between groups.

**Figure 3:**
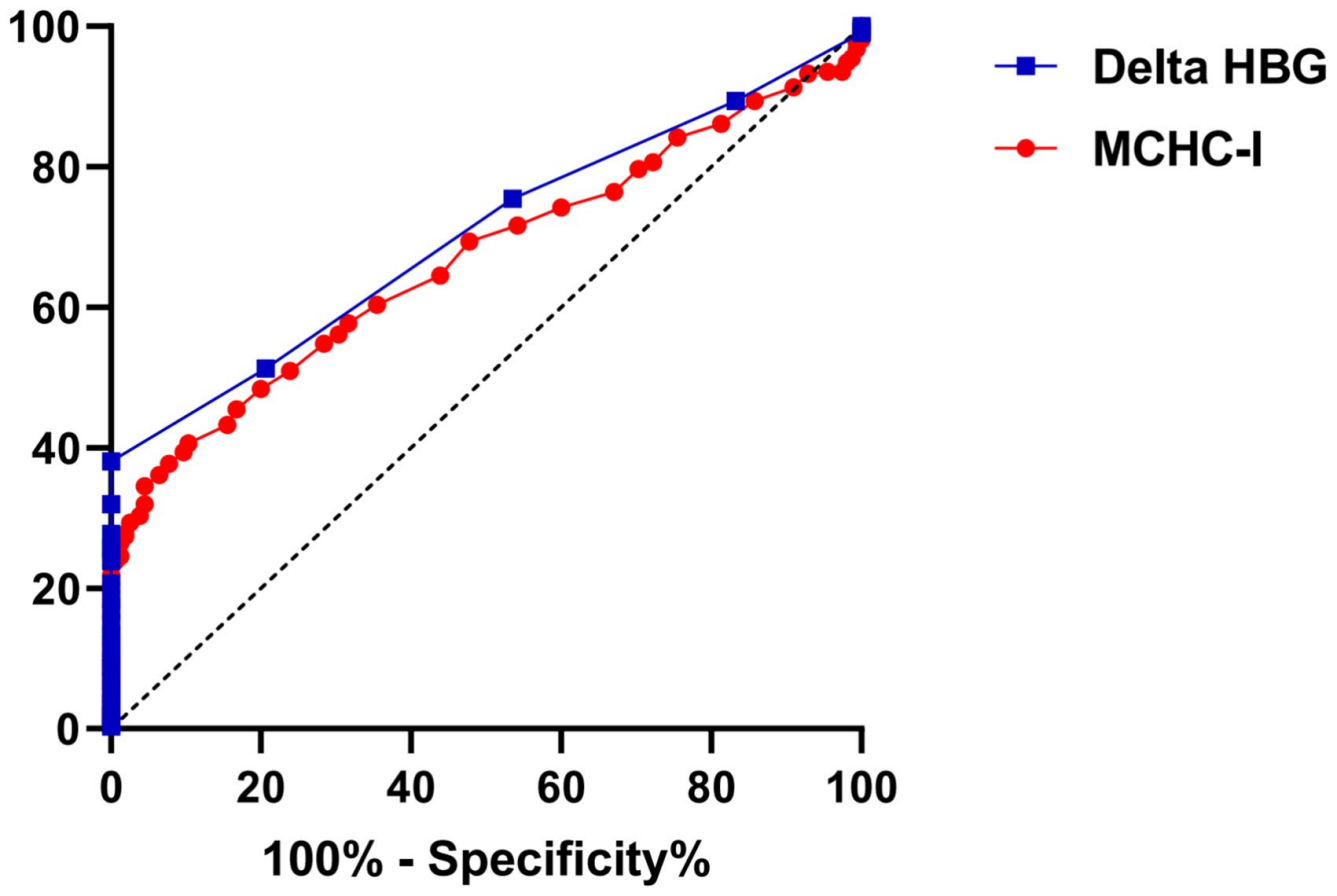
Receiver operating characteristic curves of Delta-HGB and MCHC-I for identification of lipemic interference.

Compared with the non-lipemic group, the lipemic group showed significantly higher MCHC-I (*p* < 0.01) and Delta-HGB (*p* < 0.05; Figure 2). ROC analysis demonstrated AUCs of 0.703 (95%CI:0.658–0.749) for Delta-HGB and 0.668 (95% CI: 0.620–0.716) for MCHC-I for identification of lipemic interference overall. The optimal cutoffs were 3.5 g/L for Delta-HGB and 348.5 g/L for MCHC-I, yielding sensitivity/specificity of 38.06%/100 and 40.65%/89.68%, respectively. These findings indicate limited overall discriminatory performance for all lipemic specimens combined (Figure 3).

### Comparison of corrected values obtained by optical scatter, plasma replacement, and formula-based correction

After stratification by lipemic turbidity, no significant differences were observed in corrected HGB, MCHC, or MCH among the optical scatter method, plasma replacement method, and formula-based correction method in the 1+, 2+, or 3 + groups (all *p* > 0.05; Tables 2–4). For HGB, the *p* values across the three lipemic strata were 0.7376, 0.8070, and 0.9139. The corresponding p values were 0.0565, 0.0566, and 0.0983 for MCHC and 0.3817, 0.2580, and 0.6210 for MCH. These findings indicate that optical scatter-derived corrected values are comparable to those obtained with plasma replacement and formula-based correction.

**Table 2:** Comparison of corrected HGB values obtained by three methods in lipemic samples with different turbidity grades.

HGB result	1 + (*n* = 116)	2 + (*n* = 112)	3 + (*n* = 82)
HGB-O	145.0 (129.8, 154.2)	149.0 (139.8, 162.0)	144.5 (136.0, 159.0)
Plasma replacement	146.0 (131.0, 155.0)	150.0 (139.0, 160.0)	146.0 (137.0, 159.8)
Formula-based correction	146.0 (131.8, 156.0)	151.0 (140.8, 162.0)	146.0 (137.0, 161.0)
H value	0.6087	0.4288	0.1801
*p* value	0.7376	0.8070	0.9139

**Table 3:** Comparison of corrected MCHC values obtained by three methods in lipemic samples with different turbidity grades.

MCHC result	1 + (*n* = 116)	2 + (*n* = 112)	3 + (*n* = 82)
MCHC-O	334.5 (329.0, 342.0)	337.5 (330.0, 344.2)	338.0 (334.0, 344.8)
Plasma replacement	336.0 (331.0, 343.0)	341.0 (334.8, 347.2)	341.0 (335.2, 346.8)
Formula-based correction	339.0 (336.0, 344.2)	338.0 (332.8, 345.8)	341.0 (336.2, 345.0)
H value	5.747	5.742	4.639
*p* value	0.0565	0.0566	0.0983

**Table 4:** Comparison of corrected MCH values obtained by three methods in lipemic samples with different turbidity grades.

MCH result	1 + (*n* = 116)	2 + (*n* = 112)	3 + (*n* = 82)
MCH-O	30.4 (29.7, 31.3)	30.8 (29.9, 31.4)	30.1 (29.5, 31.4)
Plasma replacement	30.2 (29.4, 31.2)	30.5 (29.3, 31.4)	30.0 (29.0, 31.5)
Formula-based correction	30.5 (29.6, 31.3)	30.7 (29.6, 31.5)	30.2 (29.3, 31.4)
H value	1.926	2.709	0.9528
*p* value	0.3817	0.2580	0.6210

**Figure 4:**
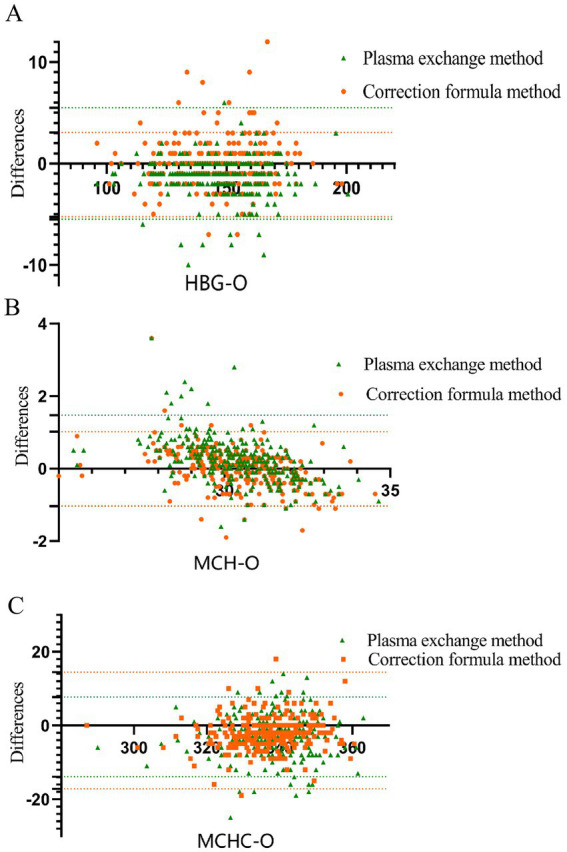
Bland–Altman agreement analysis of corrected results obtained by the optical scatter method, plasma replacement method, and formula-based correction method. **(A)** HGB. **(B)** MCH. **(C)** MCHC.

In Bland–Altman analysis, when optical scatter-based corrected HGB was compared with plasma replacement, 2.9% (9/310) of data points fell outside the 95% limits of agreement and the mean bias was 0.003. When optical scatter-based corrected HGB was compared with formula-based correction, 4.5% (14/310) of points fell outside the 95% limits of agreement and the mean bias was −1.087, indicating good agreement (Figure 4A). For MCH, 3.5% (11/310) of points were outside the 95% limits of agreement in the comparison between the optical scatter and plasma replacement methods, with a mean bias of 0.22. In the comparison between the optical scatter and formula-based methods, 3.9% (12/310) of points were outside the 95% limits of agreement and the mean bias was −0.002, indicating clinically acceptable agreement (Figure 4B). For MCHC, similar proportions were observed: 4.8% (15/310) and 0.65% (2/310) of data points fell outside the 95% limits of agreement, respectively. The corresponding mean biases were −3.071 and −1.368, both of which were considered clinically acceptable (Figure 4C).

### Influence of lipemic turbidity on HGB and related parameters

Further stratification by lipemic index showed that the impact of lipemia on HGB increased with increasing turbidity. In the 1 + and 2 + groups, HGB-I did not differ significantly from HGB-O (*p* = 0.3633 and *p* = 0.2658, respectively), whereas in the 3 + group HGB-I was significantly higher than HGB-O (*p* < 0.0001), indicating that severe lipemia can cause marked false elevation of colorimetric HGB (Table 5). MCH followed a similar pattern: no significant difference between MCH-I and MCH-O was observed in the 1 + and 2 + groups (*p* = 0.3495 and *p* = 0.1430, respectively), whereas MCH-I was significantly higher than MCH-O in the 3 + group (*p* < 0.0001) (Table 6). In contrast, MCHC differed significantly between the colorimetric and optical scatter methods in all lipemic strata, with *p* values of 0.0005, <0.0001, and <0.0001 in the 1+, 2+, and 3 + groups, respectively, suggesting that MCHC is more sensitive to lipemic interference (Table 7).

**Table 5:** Comparison of HGB-I and HGB-O in lipemic samples with different turbidity grades.

HGB result	1 + (*n* = 116)	2 + (*n* = 112)	3 + (*n* = 82)
HGB-I	147.0 (131.8, 156.0)	153.0 (141.8, 164.0)	164.0 (153.0, 174.8)
HGB-O	145.0 (129.8, 154.2)	149.0 (139.8, 162.0)	144.5 (136.0, 159.0)
*p* value	0.3633	0.2658	<0.0001

**Table 6:** Comparison of MCH-I and MCH-O in lipemic samples with different turbidity grades.

MCH result	1 + (*n* = 116)	2 + (*n* = 112)	3 + (*n* = 82)
MCH-I	30.5 (29.6, 31.4)	31.1 (29.9, 31.9)	33.3 (31.9, 34.5)
MCH-O	30.4 (29.7, 31.3)	30.8 (29.9, 31.4)	30.1 (29.5, 31.4)
*p* value	0.3495	0.1430	<0.0001

**Table 7:** Comparison of MCHC-I and MCHC-O in lipemic samples with different turbidity grades.

MCHC result	1 + (*n* = 116)	2 + (*n* = 112)	3 + (*n* = 82)
MCHC-I	339.0 (331.8, 344.0)	340.0 (333.0, 347.0)	344.0 (336.0, 349.2)
MCHC-O	333.0 (328.0, 340.0)	336.0 (330.0, 344.2)	337.5 (330.0, 344.2)
*p* value	0.0005	<0.0001	<0.0001

### Performance of Delta-HGB and MCHC-I for identifying severe lipemic interference

**Figure 5:**
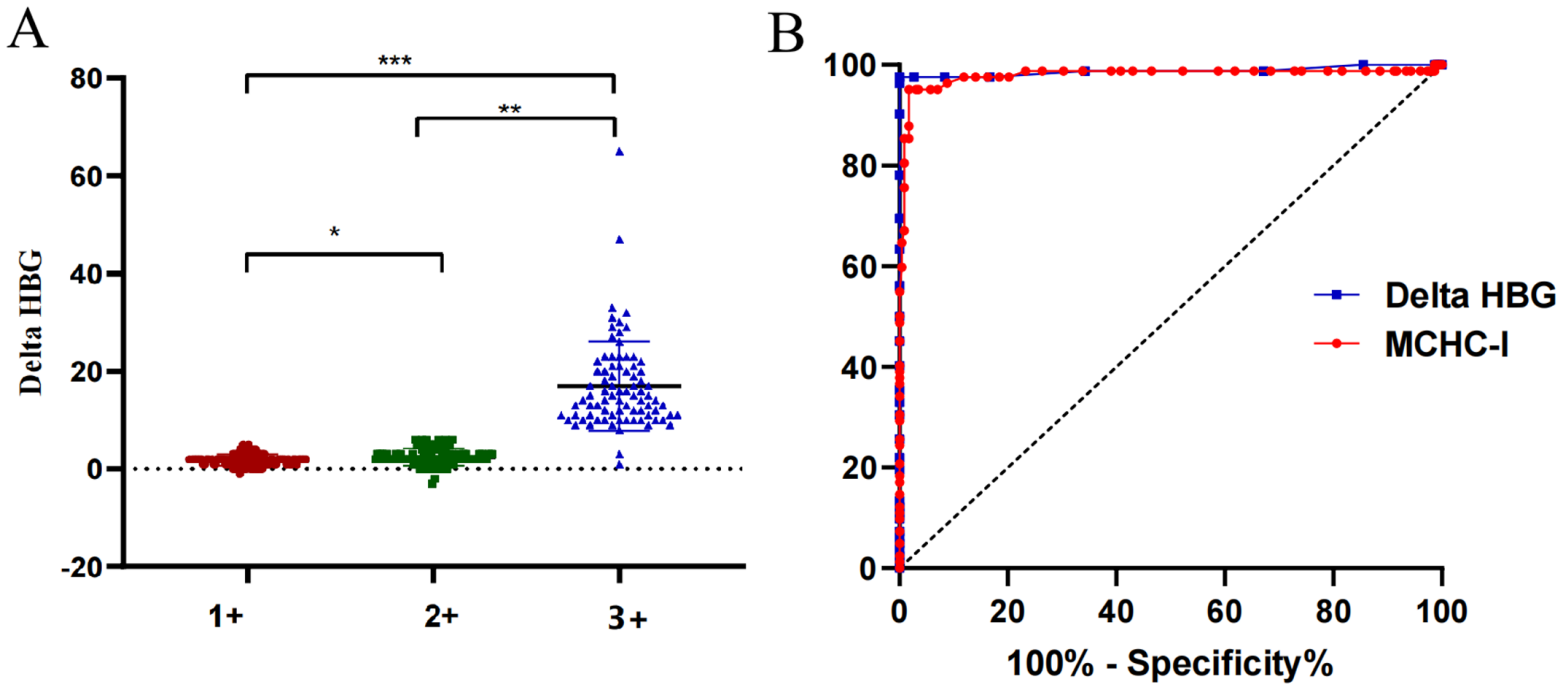
Performance of Delta-HGB and MCHC-I for identifying severe lipemic interference. **(A)** Comparison of Delta-HGB across lipemic turbidity strata. **(B)** ROC curves of Delta-HGB and MCHC-I for identifying severe lipemic interference.

When the 1 + and 2 + groups were combined and compared with the 3 + group, Delta-HGB increased significantly with increasing lipemic turbidity (Figure 5A). For identification of severe lipemic interference, ROC analysis yielded AUCs of 0.988 (95% CI:0.970–1.000) for Delta-HGB and 0.979 (95% CI:0.950–1.00) for MCHC-I, representing a marked improvement over the overall analysis of all lipemic samples. The optimal cutoffs were 7 g/L for Delta-HGB and 359.5 g/L for MCHC-I. At these thresholds, the sensitivity and specificity were 97.56 and 100% for Delta-HGB and 95.12 and 98.25% for MCHC-I, respectively. These results indicate that the combination of Delta-HGB and MCHC-I provides excellent discrimination of severe lipemic interference (Figure 5B).

## Discussion

Using real-world clinical lipemic specimens, this study evaluated the utility of optical scatter-derived red blood cell parameters generated in the RET channel of an automated hematology analyzer for both identification and correction of lipemic interference. The main findings were that the combination of Delta-HGB and MCHC-I effectively identified severe lipemic interference, and that HGB, MCHC, and MCH corrected by the optical scatter method showed good agreement with results obtained by plasma replacement and formula-based correction. These findings support the practical value of the optical scatter method in routine laboratory work.

When all lipemic samples were analyzed together against non-lipemic samples, the overall discriminatory performance of Delta-HGB and MCHC-I was modest, with AUCs of 0.703 and 0.668, respectively. This suggests limited ability to distinguish mild-to-moderate lipemic interference. However, stratification by lipemic index showed that Delta-HGB values in the 1 + and 2 + groups were close to those in the non-lipemic group, whereas values were clearly increased in the 3 + group. After combining the 1 + and 2 + groups and comparing them with the 3 + group, the AUCs increased to 0.988 for Delta-HGB and 0.979 for MCHC-I, with optimal cutoffs of 7 g/L and 359.5 g/L, respectively. These data indicate that both markers are particularly useful for identifying severe lipemic interference.

Previous studies have commonly used MCHC thresholds >360 g/L or >365 g/L as indicators of lipemic interference (3–6, 14). The optimal cutoff of 359.5 g/L observed here is therefore highly consistent with prior reports. However, elevated MCHC is not specific to lipemia and may also occur in cold agglutination, spherocytosis, and other conditions affecting red blood cell indices (3–5, 8–10, 15, 16). The present findings suggest that Delta-HGB measured in the RET channel can improve specificity when used together with MCHC-I in samples with markedly increased MCHC.

The clinical utility of RET channel-derived parameters for resolving increased MCHC has also been demonstrated in previous studies. Berda-Haddad et al. proposed a decision-tree approach using RBC-O and HGB-O obtained from the Sysmex XN-10 RET channel to distinguish analytical artifacts from pathological causes of increased MCHC, including red blood cell agglutination, optical interference, and red blood cell disorders (17). Their study provided important evidence supporting the use of optical parameters for the interpretation of abnormal erythrocyte indices. In contrast, the present study focused specifically on lipemia-induced interference and evaluated Delta-HGB, HGB-O, and MCHC-O in clinical lipemic specimens. Although the study designs and target conditions differ, our findings are consistent with the concept that RET channel-derived optical parameters can assist in identifying and correcting spurious HGB and erythrocyte index abnormalities.

An additional finding was that the effect of lipemia on HGB measurement was more closely related to specimen turbidity than to triglyceride concentration alone. For example, one sample had a TG concentration as high as 25.51 mmol/L but a lipemic index of only 1+, and the differences among the colorimetric, optical scatter, plasma replacement, and formula-based results were small. This may reflect heterogeneity in lipoprotein particle size. Larger chylomicrons contribute more strongly to turbidity and light scattering, whereas smaller very-low-density lipoprotein particles can increase TG without producing comparable visual turbidity (18). These observations support the use of lipemic index rather than TG concentration alone for stratifying the analytical impact of lipemia.

With respect to correction, no significant differences in HGB, MCHC, or MCH were found among the optical scatter, plasma replacement, and formula-based methods across lipemic turbidity strata, and Bland–Altman analysis demonstrated overall good agreement. Although a small number of data points fell outside the 95% limits of agreement, this may have been related to incomplete mixing, mild red blood cell injury during plasma replacement, or osmotic changes during specimen handling. From a clinical perspective, the observed mean biases remained acceptable, supporting the feasibility of using the optical scatter method for correction of lipemic interference.

Compared with plasma replacement and formula-based correction, the major advantage of the optical scatter method is that it does not require centrifugation, plasma replacement, or additional measurement of plasma HGB. Instead, corrected HGB and related indices can be obtained simply by switching the analyzer to the RET channel. This approach has the potential to shorten turnaround time, streamline workflow, and reduce staff workload, which is particularly relevant in the context of persistent laboratory workforce shortages (19).

It should be emphasized that isovolumetric plasma replacement is not intended for routine use in all lipemic specimens. In clinical practice, this procedure is generally most relevant for specimens with severe hyperlipemia or marked lipemic turbidity, in which lipid particles can substantially affect automated colorimetric HGB measurement and derived erythrocyte indices. In the present study, plasma replacement was used primarily as a practical comparative reference method to evaluate optical scatter-derived correction across different degrees of lipemic turbidity. Consistent with this practical consideration, significant elevation of colorimetric HGB was observed mainly in the 3 + lipemic group, whereas HGB-I and HGB-O did not differ significantly in the 1 + and 2 + groups. These findings suggest that plasma replacement may be most clinically necessary for severe lipemic interference rather than for mild or moderate lipemia.

Based on the present findings, we propose a targeted workflow for the clinical implementation of RET channel-derived parameters in specimens with increased MCHC. First, routine CBC results should be reviewed for increased MCHC-I. When MCHC-I is greater than 359.5 g/L, the sample should be reflexed to the RET channel to obtain Delta-HGB and optical scatter-derived parameters. If Delta-HGB is greater than 7 g/L, severe lipemic interference should be suspected, and HGB-O, MCHC-O, and MCH-O may be used as corrected values after standard laboratory review. If Delta-HGB is not greater than 7 g/L, clinically significant lipemic interference is unlikely, and other causes of increased MCHC, including hemolysis, cold agglutinins, hemoglobinopathies, or red blood cell abnormalities, should be considered.

To preliminarily verify the feasibility of this workflow, we additionally tested 20 randomly retained CBC samples with MCHC-I greater than 359.5 g/L. Among these samples, three had Delta-HGB greater than 7 g/L, and all three showed creamy-white plasma after centrifugation, consistent with severe chylous lipemia. The remaining 17 samples had Delta-HGB not greater than 7 g/L; after centrifugation, three showed cherry-red plasma consistent with marked hemolysis, and 14 showed clear plasma. These findings support the practical value of combining MCHC-I with Delta-HGB for identifying severe lipemic interference in samples with increased MCHC.

This study has several limitations. First, this was a retrospective study based on available residual clinical specimens, and no prospective sample size or power calculation was performed. Second, this was a single-center study conducted using one hematology analyzer platform, which may limit the generalizability of the findings to other laboratories, patient populations, and instrument systems. Third, no external validation cohort was available, and the number of samples in the severe lipemia subgroup was relatively small, which may affect the robustness of the stratified analysis and proposed cutoffs. Finally, a multivariate prediction model combining Delta-HGB and MCHC-I was not established in the present study. Future multicenter studies with larger sample sizes, different analyzer platforms, and independent validation cohorts are needed to confirm these findings and further optimize the diagnostic workflow.

## Conclusion

In summary, the combination of Delta-HGB and MCHC-I is an effective indicator set for identifying severe lipemic interference. The optical scatter method can correct lipemia-induced bias in HGB and related erythrocyte parameters without specimen pretreatment and offers a simple, rapid, and accurate approach with promising value for broader implementation in clinical laboratories.

## Data Availability

The original contributions presented in the study are included in the article/supplementary material, further inquiries can be directed to the corresponding author.
